# Editorial: Computational biomechanics for ventricle-arterial dysfunction and remodeling in heart failure, Volume II

**DOI:** 10.3389/fphys.2022.1100037

**Published:** 2022-12-08

**Authors:** Yunlong Huo, Shaun D. Gregory

**Affiliations:** ^1^ Institute of Mechanobiology and Medical Engineering, School of Life Sciences and Biotechnology, Shanghai Jiao Tong University, Shanghai, China; ^2^ PKU-HKUST Shenzhen-Hong Kong Institution, Shenzhen, Guangdong, China; ^3^ Cardio-Respiratory Engineering and Technology Laboratory, Department of Mechanical and Aerospace Engineering, Monash University, Melbourne, VIC, Australia

**Keywords:** computational biomechanics, heart failiure, hemodynamics, FFR = fractional flow reserve, soft tissue mechanical properties

## Introduction

Interactions between the left ventricle (LV) and systemic circulation, and between the right ventricle (RV) and the pulmonary circulation are key determinants of cardiac and cardiovascular function. The global performance of LV-arterial coupling (e.g., the ratio of effective arterial elastance, time-varying pressure-flow relations, and effects of wave reflections) has been applied to many clinical scenarios such as aging, hypertension, heart failure (HF), and dilated cardiomyopathy. The global approach, however, has significant limitations in heart failure with preserved ejection fraction (HFpEF), although it can provide useful information regarding the mechanical efficiency and performance in heart failure with reduced ejection fraction (HFrEF). This approach is also less informative in the study of RV dysfunction and its coupling with the pulmonary circulation in HF. With the development of simulation-based biomechanics in recent years, it is required to demonstrate accurate analysis of local ventricle-arterial functions and remodeling in health and disease, particularly in the progression of HF.

This Research Topic in computational biomechanics was conceived to improve our understanding and treatment of cardiac and cardiovascular dysfunction and their coupling abnormalities associated with the occurrence and development of HFpEF or HFrEF. There is a total of 12 published articles (11 original research and 1 brief research report) relevant to basic and clinical studies covering: 1) cardiac stress and strain analysis using computational models; 2) hemodynamics in systemic/pulmonary circulations or coronary circulation; 3) advanced biomechanics models of LV/RV-arterial coupling and remodeling; 4) computational models based on bio-imaging measurements in patients of HF; 5) machine learning methods to enhance the accuracy of computational biomechanics; and 6) computational models to aid in the development of medical devices for treatment of HF. Here, we briefly summarize the contributions from the 12 publications.

### Computational simulation of heart valves and cardiovascular devices

Computational analysis of the native cardiovascular system, and devices that are used to treat the diseased native cardiovascular system, is regularly conducted to enhance our understanding of patient anatomy and blood flow dynamics (Neidlin et al., 2021; Vatani et al., 2022). This enhanced understanding can lead to improvements in patient management, better cardiovascular devices, and additional applications of existing devices. Modern cardiovascular computational simulations have progressed beyond simplified geometries to patient-specific models based on medical imaging, with some progressing further to statistical shape models based on larger cohorts of patient data (Goubergrits et al., 2022).

In this Research Topic, three-dimensional statistical shape models of healthy patients and those with varying degrees of tricuspid valve regurgitation were generated from cardiac MRI data (Orkild et al.). Those with tricuspid regurgitation demonstrated increased right ventricular free wall bulging, narrowing of the base, and blunting of the right ventricular apex. Compared with tricuspid regurgitation, aortic stenosis is even more common, with low-flow and low-gradient aortic stenosis (LFLG AS) being a commonly recognized sub-type with controversial management strategies due in part to a lack of biomechanics knowledge. To improve the knowledge of LFLG AS biomechanics, a patient-specific model using ECG-gated cardiac computed tomography was developed using a previously validated multi-scale, multi-physics computational heart model coupled with a virtual circulatory system and calibrated to clinically-measured parameters in this issue (Wisneski et al.). Progression of valvular disease may necessitate placement of a mechanical replacement valve, with minimally invasive transcatheter aortic valve replacement (TAVR) increasing in popularity. In this issue, a fluid-structure interaction model of TAVR was used to investigate blood flow dynamics, revealing variations in maximum leaflet stress, opening area, and low-velocity areas when leaflet geometries were altered (Liu et al.).

Cardiovascular modelling techniques can combine blood flow simulations with structural models (e.g. fluid-structure interaction models), lumped parameter modelling, and even *in-vivo* experiments. In this issue, a combination of cardiac electrical signals, blood pressure and echocardiography imaging from a pacing animal model was combined with a cardiac fluid-structure interaction model to demonstrate the influence of pacemaker location on cardiac outcome (Fan et al.). In another research topic, a combination of patient images *via* computed tomography and lumped parameter modelling was used in this issue to investigate the influence of atrial fibrillation and left atrial appendage occlusion on thrombosis in a simulated patient supported by a left ventricular assist device (Ghodrati-Misek et al.). Results obtained from the advanced contractile left-heart model revealed unfavorable left atrial flow dynamics during atrial fibrillation which was improved after left atrial appendage occlusion, which may reduce the potential for thrombus formation within the left atrium.

### Computational techniques for physiological assessments

Fractional flow reserve (FFR) is the Class Ia recommendation for guiding the decision to revascularize epicardial coronary stenoses by societal guidelines in Europe and United States (Knuuti et al., 2020; Writing Committee et al., 2022). Some practical restrictions limit the traditional wire-based FFR utilization, such as requiring drug-induced hyperemia, pressure drift of the pressure wire, and so on (Gong et al., 2020; Li et al., 2020; Gong et al., 2022). Multiple computational techniques have been developed to determine FFR based on computed tomography angiograms (CTA-FFR) (Taylor et al., 2013). The diagnostic accuracy of CTA-FFR, however, varied significantly across the spectrum of cardiovascular diseases (Cook et al., 2017). Hence, it is still required to improve the computational techniques. In this Research Topic, a method for calculating FFR based on steady-state geometric multiscale (FFR_SS_) was proposed based on a coronary artery model segmented from a patient’s coronary CTA images (Liu et al.). The diagnostic performance of FFR_SS_ and traditional FFR_CT_ was compared with the wire-based FFR. FFR_ss_ showed similar accuracy to FFR_CT_, but improved the calculation efficiency. Moreover, a fluid–structure interaction (FSI) algorithm with a physics-driven 3D–0D coupled mode was developed to improve the accuracy of CTA-FFR (Xi et al.). This method improved the diagnostic accuracy of CTA-FFR computation.

The coronary arterial trees include millions of blood vessels, most of them are small arterioles and pre-capillary vessels (Huo et al., 2009). An index of microcirculatory resistance (IMR) was proposed to quantify coronary microcirculatory dysfunction (Fearon et al., 2003; Aarnoudse et al., 2004; Fearon et al., 2004). However, its application within clinical practice remains extremely limited due to a complex guide wire measurement under hyperemia (Ai et al., 2020). Coronary angiography-derived IMR (caIMR) has been proven to have high correlation and diagnostic accuracy with invasive wire-based IMR (Ai et al., 2020; Choi et al., 2021). In this Research Topic, caIMR >40 was shown to be an independent predictor of the combined events including cardiovascular death or heart failure readmission and hence a promising method for prognosis in STEMI patients (Duan et al.).

### Hemodynamics and vessel wall mechanics

Various abnormal hemodynamic parameters such as low wall shear stress (WSS), high oscillatory shear index (OSI), old blood volume fraction (OBVF), and old blood volume (OBV), have been proposed to investigate the occurrence and development of atherosclerosis (Huang et al., 2016; Fan et al., 2017; Feng et al., 2020). In this Research Topic, thrombosis risk was evaluated within occluded coronary arterial fistulas (CAF) with terminal aneurysms using untreated, aneurysm-reserved and aneurysm-removed numerical models (Jiang et al.). The OBV was found to be superior to the area of high OSI and low WSS in determining treatment type. On the other hand, competitive flow and anastomotic stenosis are two risk factors for poor instant patency of coronary artery bypass grafting (CABG) surgery (Fan et al., 2016; Fan et al., 2017). A 0D-3D coupled multiscale CABG model was developed to investigate anastomotic stenosis and competitive flow (Mao et al.). The graft flow waveform shape and flow fast Fourier transformation (FFT) ratio were found to predict the poor instant patency after CABG.

In vessel wall mechanics, wall tissue fatigue is a chronic failure process induced by repetitive loading and could impact plaque development under pulsatile blood pressure (Guo et al.). In this Research Topic, the relationship between fatigue and stenosis progression was investigated based on *in-vivo* intravascular ultrasound (IVUS) images and finite element models (Guo et al.). Stenosis progression was associated with the maximum stress amplitude, average stress amplitude and average strain amplitude.

In comparison with fine particle pollution (PM2.5), ultrafine particles (UFPs) (PM0.1) produce stronger chemical reactions given their small volume and large surface area (Li et al., 2021; Huo and Li). In this Research Topic, the change of cardiac function and peripheral hemodynamics was investigated in rats of myocardial infarction (MI) after long-term inhalation of ultrafine Zn particles (Huo and Li). The long-term inhalation of ultrafine zinc particles induced excessive accumulation of zinc in serum and tissue, which deteriorated cardiac and hemodynamic dysfunctions in MI rats.

In summary, this special edition incorporates novel cardiovascular modelling research into a single issue to advance the knowledge in cardiovascular disease, simulation techniques, medical devices, and more.
